# Dataset of 2012-2020 U.S. National- and State-Level Greenhouse Gas Emissions by Sector

**DOI:** 10.1016/j.dib.2024.110173

**Published:** 2024-02-16

**Authors:** Ben Young, Catherine Birney, Wesley W. Ingwersen

**Affiliations:** a Eastern Research Group, Inc. 561 Virginia Rd Suite 300 Building 4, Concord, MA 01742 USA; b Center for Environmental Solutions and Emergency Response, Office of Research and Development, US Environmental Protection Agency, 26 W. Martin Luther King Drive, Cincinnati, OH 45268 USA

**Keywords:** Environmental-economic model, Sector attribution model, FLOWSA, GHG inventory

## Abstract

The dataset contains ∼1.1 million records of total greenhouse gases directly emitted annually by economic sectors and households in the US from 2012-2020. Data are given for 16 unique greenhouse gases by 118 aggregate sectors for each state, and as totals by these aggregate sectors as well as by 540 detailed sectors at the national level. The dataset is a product of updated sector attribution models that improve upon the National Greenhouse Gas Industry Attribution Model. This paper provides documentation of the methods used to produce these datasets and proof of validation of the dataset, along with relevant supporting tables, figures, and source code.

Specifications Table

**Table:** 

Subject	Pollution
Specific subject area	Environmental-economic accounting of greenhouse gas emissions
Data format	Analyzed
Type of data	Table
Data collection	Data are modeled by combining several sources. The modeling approach is flow sector attribution modeling where flows such as greenhouse gas (GHG) emissions are attributed or allocated to economic sectors based on direct emissions reports or other sector-level activity data. The primary source of annual flows is the U.S. Greenhouse Gas Inventory. Secondary sources for attribution include the disaggregated state-level GHG emission inventories, the Manufacturing Energy Consumption Survey, U.S. Supply/Make and Use input-output tables, employment data and various other minor sources.
Data source location	United States
Data accessibility	Repository name: Data.gov Data identification number: DOI: 10.23719/1529805 Direct URL to data: http://doi.org/10.23719/1529805

## 1 Value of the Data

• Tracking greenhouse gas emissions is important for governments and organizations seeking to estimate progress in reducing the drivers of climate change-related impacts.
• While the U.S. GHG Inventory (GHGI) is an essential resource for understanding the total annual GHG emissions attributable to the U.S., it does not fully attribute these emissions to economic sectors at a highly detailed resolution, which is the purpose of the modeling and resulting datasets described here.
• Researchers looking to assess U.S. greenhouse gas emission trends associated with sector/industry activities will find these data valuable.
• The data can be used in sector- and national-scale models of GHG emissions associated with economic activity including environmentally-extended input-output models, life cycle assessment models, or to produce environmental-economic accounts for GHGs for the U.S.

## 2 Background

This dataset provides an update to the output of what was originally the National Greenhouse gas Industry Attribution Model (NGIAM) [1]. The model attributes GHG emissions (more generically flows for sector attribution model) from primary data sources to specific industries, where not all flows are at the target level of industry detail or not given by the industry classification that is required, and hence additional sources are used to attribute or allocate the flows to a given industry. The most recent NGIAM was published in 2020 [1] in association with the Supply Chain GHG Emission Factors [2]. The 2020 NGIAM is an Excel © model that provides totals of GHGs for years 2010-2016 by industries as defined in the 2012 BEA Detailed Input-Output tables [3], which are aggregations of 2-to-6 digit North American Industry Classification System (NAICS) codes. The objective of this work was to update the data and architecture of NGIAM model to create a dataset of GHG emissions by U.S. industries. This document also improves upon the documentation of the NGIAM, which has not been previously described in a peer-reviewed format.

## 3 Data Description

The 2012-2020 Greenhouse Gas (GHG) National- and State-Level Emission Totals By Sector dataset [4] contains ∼1.1 million records of GHGs directly emitted in the U.S. by economic sectors and households across three Excel © files. The data provide greenhouse gas totals by sector across a U.S. state for a given year at an aggregate level with 118 unique sectors. At the national scale, totals by sector are provided at the same aggregate industry level as well as at a more detail level, with 540 unique U.S. sectors. Sectors are defined using the sector code from the 2012 version of the NAICS. The 2022 release of the U.S. GHG Inventory (GHGI) is the primary source the emissions quantities in the dataset [5]. The 16 unique GHG chemicals or groups are reported using the nomenclature from the Federal LCA Commons Elementary Flow List v1.2.0 [6], where all individual GHGs present in the GHGI are reported. The dataset uses the Flow-By-Sector collapsed format [7]. Emissions of each GHG are reported in kilograms (kg) per year. One exception is groups of hydrofluorocarbons (HFCs) where the original data were only available in CO_2_-equivalents (CO2e). The geographic boundary includes the 50 U.S. states and the District of Columbia. Even more detailed versions of the Excel datasets are available in Apache parquet files, where each record provides a total for a given GHG, sector, year, location, and data source and provides all the fields in the Flow-By-Sector collapsed format. The Excel versions of the data provide records aggregated at the level of GHG, sector, year, and location. The Excel versions also provide supporting definitions of sector codes.

The first 15 rows of the full tables (Apache parquet format) for detailed sector-level national values for year 2020 are shown in Table 1. The first three records show CO_2_ emissions for sector 11111, which is soybean farming, from three different sources, including applying lime to fields, applying urea fertilizer, and combustion of petroleum in farming operations. The data come from the EPA GHGI tables 2.1 and A5. Users can refer to the description of the data in the GHGI for more information on the emission source.

**Table 1:** First 15 records for 2020 detailed sector estimates in dataset.

Flowable	Sector	Context	FlowAmount	MetaSources	AttributionSources
Carbon dioxide	11111	emission/air	1200000000	EPA_GHGI_T_2_1.liming	Detail_Use
Carbon dioxide	11111	emission/air	290732098	EPA_GHGI_T_2_1.urea_fertilizer	Detail_Use
Carbon dioxide	11111	emission/air	1029056448	EPA_GHGI_T_A_5.petroleum	Detail_Use
Methane	11111	emission/air	17309	EPA_GHGI_T_3_8.fuel_oil	Detail_Use
Nitrous oxide	11111	emission/air	4356	EPA_GHGI_T_3_9.fuel_oil	Detail_Use
Nitrous oxide	11111	emission/air	64281294	EPA_GHGI_T_5_17.cropland	USDA_CoA_Cropland
Nitrous oxide	11111	emission/air	22213960	EPA_GHGI_T_5_17.fertilizer_use	Detail_Use
Nitrous oxide	11111	emission/air	6636069	EPA_GHGI_T_5_18.fertilizer_use	Detail_Use
Carbon dioxide	11112	emission/air	1200000000	EPA_GHGI_T_2_1.liming	Detail_Use
Carbon dioxide	11112	emission/air	290732098	EPA_GHGI_T_2_1.urea_fertilizer	Detail_Use
Carbon dioxide	11112	emission/air	1029056448	EPA_GHGI_T_A_5.petroleum	Detail_Use
Methane	11112	emission/air	17309	EPA_GHGI_T_3_8.fuel_oil	Detail_Use
Nitrous oxide	11112	emission/air	4356	EPA_GHGI_T_3_9.fuel_oil	Detail_Use
Nitrous oxide	11112	emission/air	2818950	EPA_GHGI_T_5_17.cropland	USDA_CoA_Cropland
Nitrous oxide	11112	emission/air	22213960	EPA_GHGI_T_5_17.fertilizer_use	Detail_Use

*Note:* The fields are defined in the Flow-by-Sector format. The MetaSources pattern is the EPA GHGI Table name following by the activity name in the table corresponding with the data used. The AttributionSources ‘Detail Use’ is another model described below; USDA_CoA_Cropland are Cropland data from the Census of Agriculture [8,9]. Names for the sectors that correspond to the codes can be found in the dataset. Columns with identical values across all records are omitted here.

**Fig. 1:**
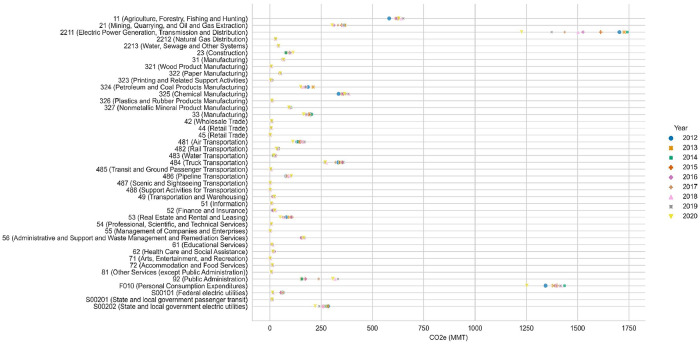
GHG totals by sector, 2012-2020, in CO2e using AR5-100 GWPs.

**Fig. 2:**
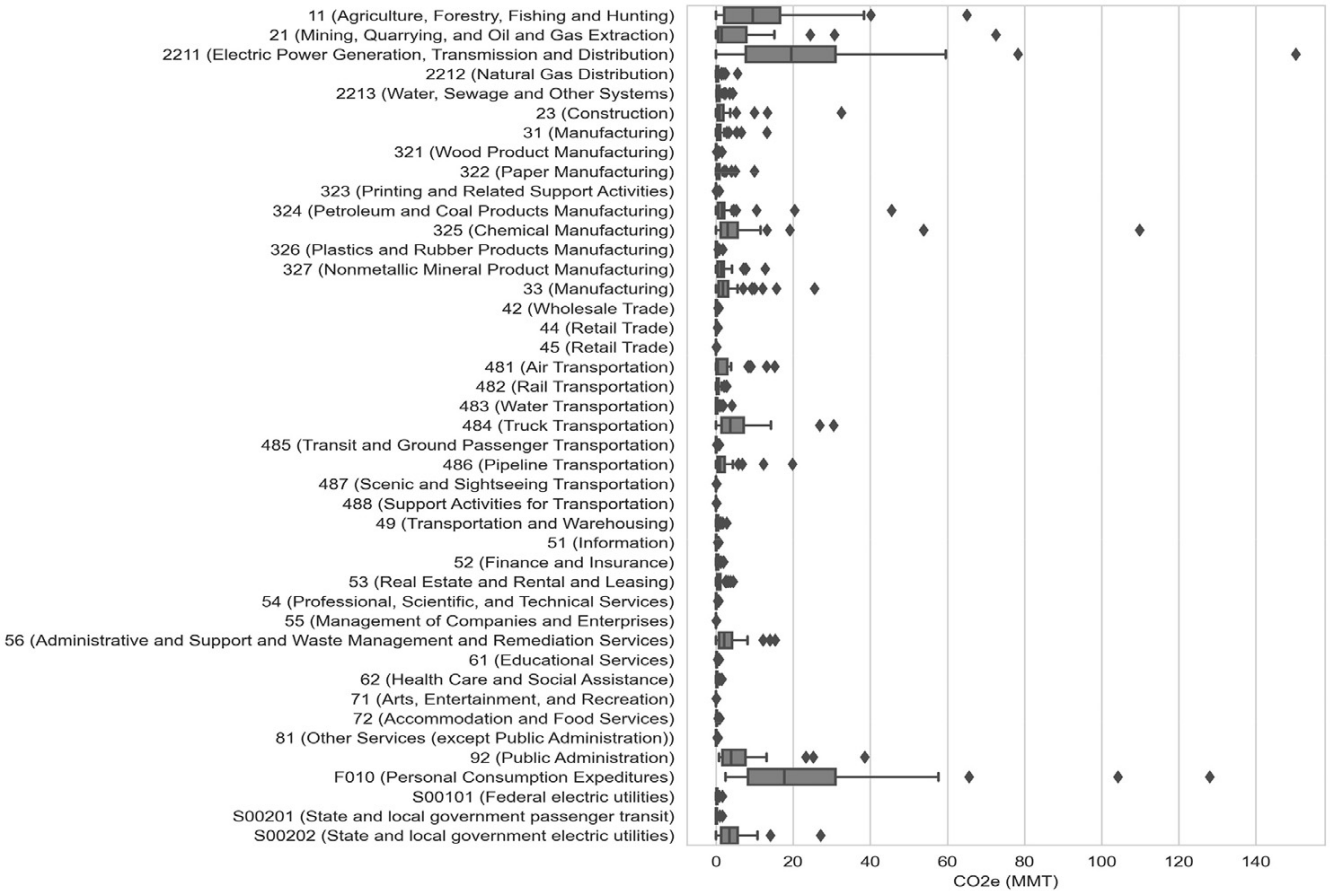
GHG totals by state and sector for 2020, presented in CO_2_-equivalent for summary purposes.U.S. totals by sector for 2020 for the three largest global warming contributors and the other GHGs combined are presented in Fig. 3 for the national aggregate data. Analogous figures produced for the U.S. detailed dataset are available on figshare[11].

For the summary tables and figures shown here, we have converted the GHG emissions in the datasets into carbon dioxide equivalents (CO2e) using estimates of their potential impact on global warming. We provide a related dataset, IPCC AR4, AR5, and AR6 20-, 100-, and 500-year GWPs [10], containing the most frequently used global warming potentials (GWPs) from the International Panel on Climate Change 4th, 5th, and 6th Assessment Reports that are aligned for use with this dataset or others using the Federal LCA Commons nomenclature. The 5th Assessment report 100-year (AR5-100) GWPs from the aforementioned dataset are used to aggregate the GHGs in our dataset to provide totals in CO2e and simplify presentation of the aggregate national models for 2012-2020 in Fig. 1 and the 2020 state models in Fig. 2. We also further aggregated sectors in these figures where GHG totals were too low to discern any differences or trends. High resolution versions of these figures along with analogous figures produced for the U.S. detailed dataset and additional lists of sector names are available on figshare [11].

**Fig. 3:**
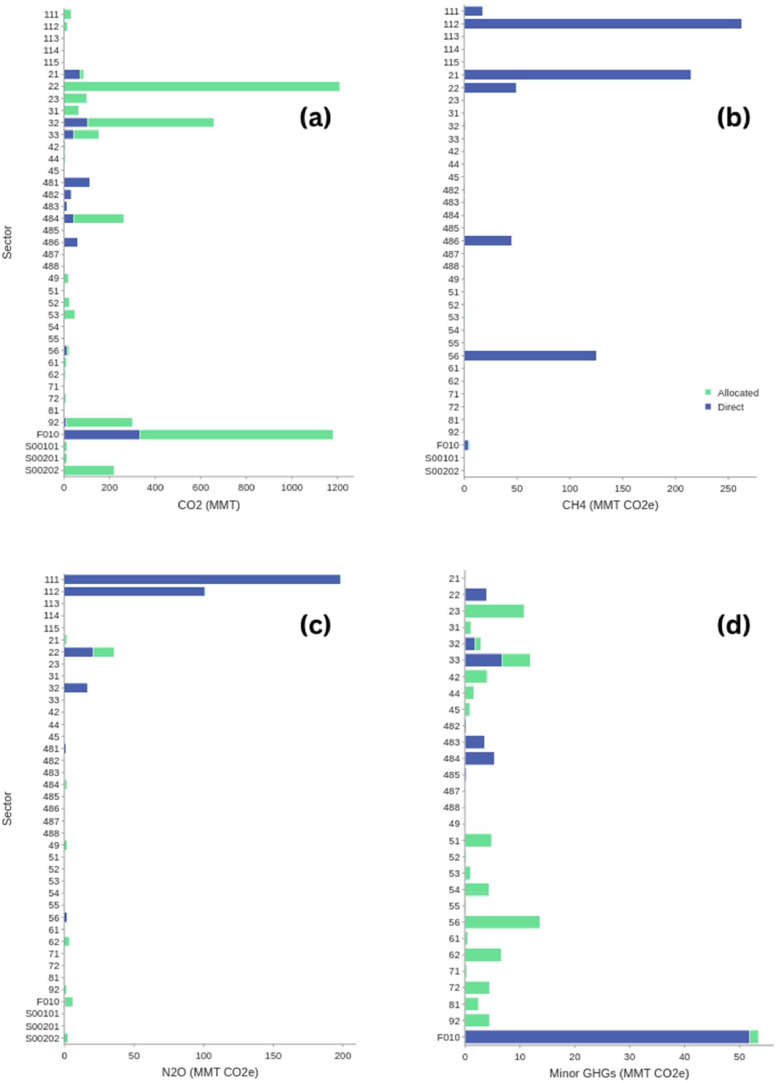
U.S. GHG emissions by aggregate NAICS sector: (a) CO_2_; (b) CH_4_; (c) N_2_O; (d) other GHGs. Each emission by sector is split into emissions that are directly attributed and emissions that are allocated. The presentation in these figures reveals quantities of GHGs that are directly attributed to industries without allocation, and those amounts that are attributed after allocation. The three largest CO2 emitting sectors groups are the electricity (221) and transport (48), and households (F010). The three largest CH4 emitting sector groups are animal agriculture (112), mining (211), waste management (562). The three largest N2O emitting sector groups are crop agriculture (111), animal agriculture (112), and electricity (221). Emissions of other GHGs, such as HFCs, fluorocarbons, sulfur hexafluoride (hereafter noted as “minor GHGs”) are more dispersed across a wide range of sectors. While most of the CO2 and N2O emissions are attributed using allocation approaches, most CH4 emissions are directly attributed. Minor GHGs from certain manufacturing and transportation sectors and households are directly attributed; however, they must be allocated to other manufacturing and service sectors.

## 4 Experimental Design, Materials and Methods

Multiple flow sector attributions models [7], also referred to as Flow-By-Sector (FBS) models, were created to produce the datasets.

**Fig. 4:**
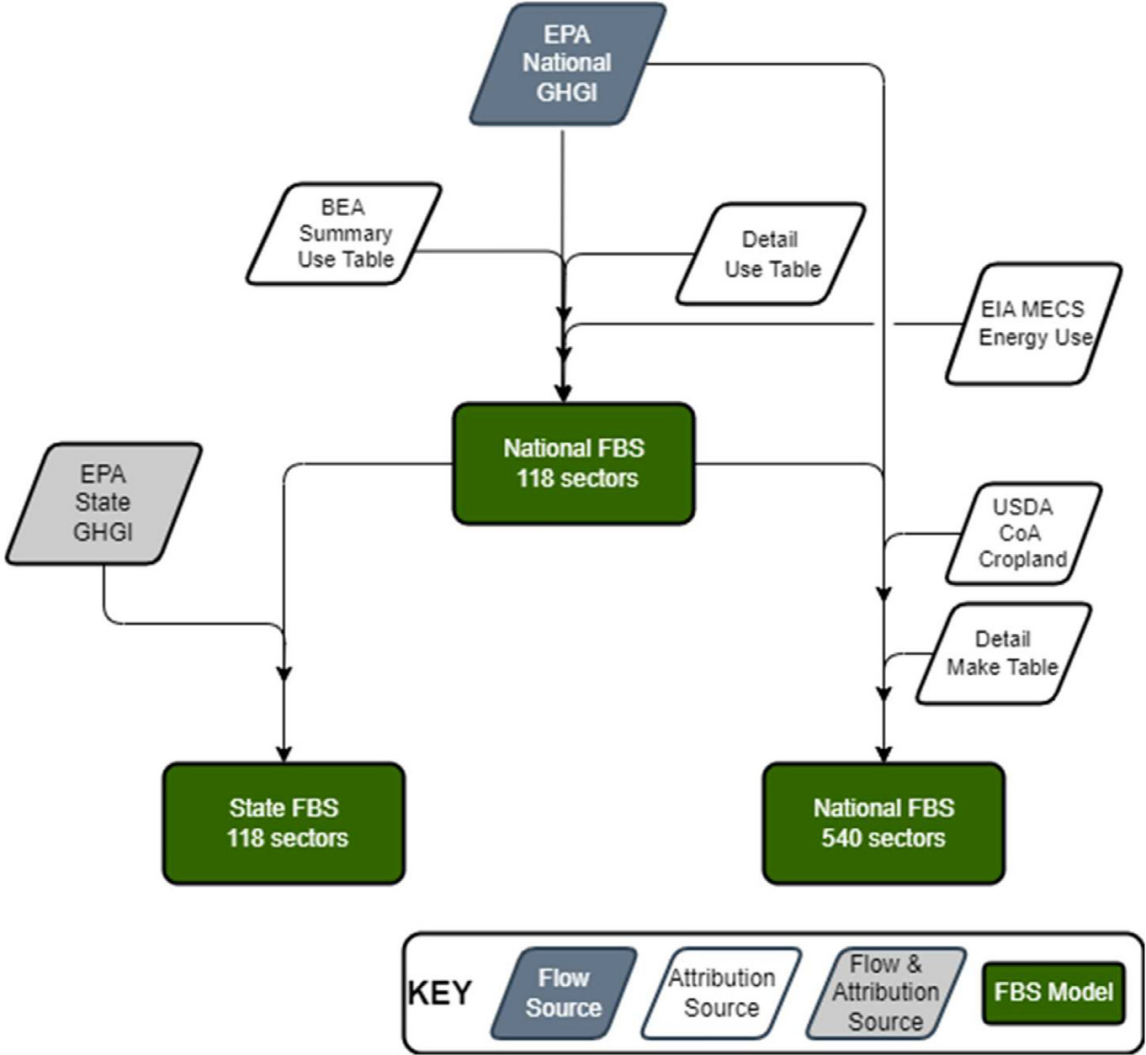
Data flow diagram for creation of aggregate and detail level national models and aggregate state level model.

The models used to generate this dataset were created in the Flow Sector Attribution (FLOWSA) tool v2.0.0 [7,12]. The modeling approach is generally consistent across data years, with only slight variations in data years represented in attribution data sources or source table or activity name changes, in keeping with changes in the annual GHGI report. The data flow through the models is represented in Fig. 4.

The creation of an aggregate level 118 sector national model is the first step. This model is a parent model for both a more detailed national-level model, and an aggregate state-level model. This parent-child relationship between the models assures consistency in calculated GHG totals by sector at the more refined industry and geographic resolutions given in the child models. The 2022 release of the GHGI is the primary emissions source for all models and is a direct input into the national aggregate model [4]. This GHGI release covers emissions from 1990-2020, although only data from 2012 to 2020 are used, which are the years available for the other datasets. The GHGI includes 100s of tables of emissions data describing various activities. GHG emissions data from these tables are carefully selected to choose emissions with activities associated with the most specific industries as possible to avoid the need for allocation. For example, emissions from Natural Gas Systems are reported by stage in Table 3-68 of the GHGI, as opposed to in aggregate in Table 2-1, which allows for attribution to specific activities of the natural gas supply chain directly, such as extraction and transmission. Emissions from 147 unique activities found in 25 unique GHGI tables are selected. Each of the selected GHGI tables are downloaded directly and converted into flow-by-activity (FBA) datasets, where records from the tables are translated into FLOWSA-defined FBA fields. For example, the year 2020 GHGI Table 2.1 indicates 0.5 CO2 MMT CO2e for Lead Production. In this example, “EPA Table_2_1” is the Source Name, “Lead Production” is the Activity-Produced-By, “CO_2_” is the Flow Name, “0.5” is the Flow Amount, and “MMTCO2e” is the Unit. Additional information is added to the FBA, such as “Chemicals” as the Class of the flows, “ELEMENTARY_FLOW” as the Flow Type, and “air” as the compartment the flow is emitted to. Metadata are also collected and stored for each FBA that includes the acquisition location (URL), date, and the githash identifier of the FLOWSA source code used to acquire and store the FBA. In a similar procedure, FBAs are also created and stored for each of the data sources used to perform attribution.

Crosswalks are created between the activities in the selected GHGI tables and the 2012 industry codes [13]. The crosswalks map an activity to the most appropriate NAICS sector(s) which could be an aggregate 2-digit sector or a more detailed 3, 4, 5, or 6-digit sector. This crosswalk is referred to in FLOWSA as an Activity-to-Sector mapping file. The crosswalks link an activity to one or more NAICS codes, where the level of resolution (from 2-6 digits) of the NAICS industry is selected based on the appropriate fit, where some activities can best be described as matching more general industries (e.g., NAICS 31-33 ‘Manufacturing’), whereas other activities can be mapped to more specific industries (e.g., NAICS 331410 ‘Nonferrous Metal (except Aluminum) Smelting and Refining’). Where an activity is linked to more than one sector in the crosswalk, or where the model specifies a higher resolution of industries (e.g., 6-digit NAICS) than specified in the crosswalk (e.g. 4-digit NAICS), attribution is required to split the GHG emissions from the original source activity across the target sectors. Crosswalks are also created for all the datasets used for attribution, where they are not already reported as NAICS codes.

The rules for attribution, which specify the flow source tables (as FBAs) that are used, which activities from the sources to use, and how to allocate them where relevant, are encapsulated in Flow-by-Sector (FBS) method files stored in FLOWSA. These method files are written in YAML, a simple human and machine-readable text format using a key-value like format. The primary method for the first step, creation of the aggregate national model, is captured in a common YAML file, *GHG_national_m1_common.yaml* and year-specific YAML files (e.g., *GHG_national_2016_m1*, the year-specific file for the 2016 data year implementation) are included that inherit all the instructions from *GHG_national_m1_common.yaml* but provide year-specific customization of flow source and attribution source data years. The Appendix provides an annotated excerpt of the *GHG_national_m1_common.yaml*. The subsequent state aggregate method includes *GHG_state_m1_common.yml* and associated year-specific YAML files, and the national detail method can be found in *GHG_national_m2_common*, also associated with year-specific YAML files. Note that “m1” and “m2” are simply notations meant to distinguish alternate methods which cover the same emissions scope.

Table 5, found in the Appendix, summarizes to which sectors GHGs from each activity are attributed and lists the data sources used when the GHGs from the activity require attribution. There are 25 unique data tables from the GHGI from which GHG emissions data are drawn. Table 5 does not specify the GHGs used from each table, and there are cases where certain gases are drawn from one table, and others from another table for the same activity. Those details can be found in *GHG_national_m1_common.yaml*.

The primary attribution sources are the same as used in the 2020 NGIAM, although data source years are updated and/or more recent releases (data updates) of the source data are used.

### 4.1 General Approaches to Allocation

When allocation is required because a higher level of industry detail is needed than provided in the data source, the allocation method is primarily proportional allocation based on a secondary flow (e.g., expenditures on some sector input). The generalized equation for proportional allocation for sector attribution modeling is shown in Eq. (1), where $e_{s}$ is the flow total for the sector s of interest, $e_{t}$ is the flow total across all sectors, $\alpha_{t}$ is the allocation flow total across all sectors, and $\alpha_{s}$ is the allocation flow for the sector of interest.

(1) $$e_{s}=e_{t}\frac{\alpha_{s}}{\alpha_{t}}$$

This can be illustrated with an example from the aggregated national model for the attribution of methane emissions from combustion for the activity “Agricultural Equipment Non-Road” from GHGI table 3.14 where the Detailed Use (henceforth “Detail Use”) table is used as the attribution source. In this case, the sector of interest is any of a number of industries that purchase Farm Machinery, for instance Oilseed Farming. A row from the Detail Use table containing dollars of industry purchases of the Farm Machinery commodity is used for the allocation. $\alpha_{s}$ in Eq. (1) would be the purchases of Farm Machinery commodities by the Oilseed Farming industry, and $\alpha_{t}$ would be the sum of all purchases across the industries of Farm equipment. The dollar units cancel out leaving a fraction, representing a relative purchases of farm machinery in relation to other agricultural sectors (as a proxy for agricultural non-road equipment) by Oilseed Farming, which is the multiplied by the total methane flow amount from “Agricultural Equipment Non-Road.” When the attribution source still does not provide information specific enough to achieve the target level of allocation, a second attribution source may be provided.

**Fig. 5:**
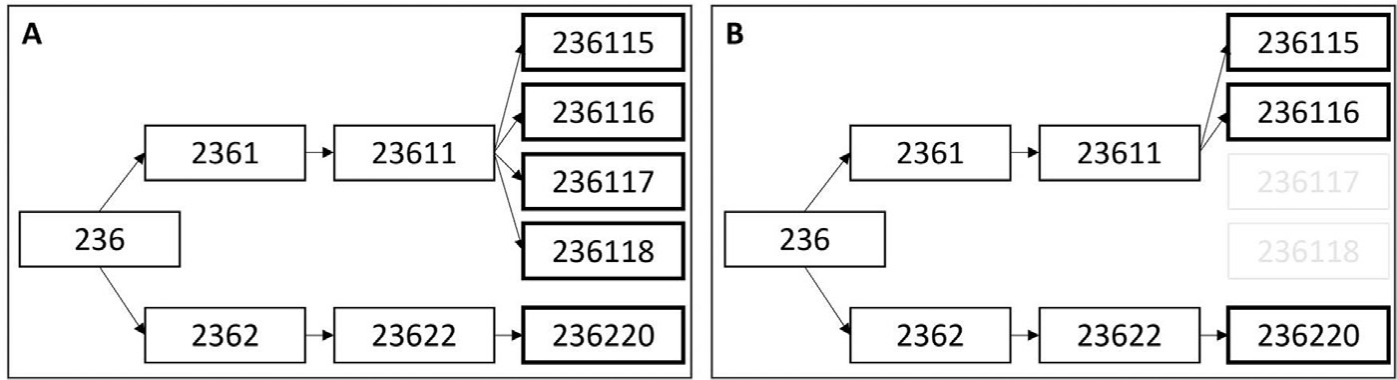
Equal allocation by sector length.

Where an attribution source cannot be identified to provide enough detail to provide flows for the target level of resolution, then equal allocation is used to allocate the remainder of the unallocated flow. In these cases, if an activity is mapped to multiple sectors, the flow amount is first equally divided across the relevant sectors. Then, the flows are equally attributed to child NAICS, based on sector length, until reaching the target sector levels. For example, if an activity with a flow amount of 16 is mapped to industries 236115, 236116, 236117, 236118, and 236220, a flow amount of 8 will be attributed to 236220 and a flow amount of 2 each to 236115, 236116, 236117 and 236118 (Box A in Fig. 5). Notably, if 236117 and 236118 were dropped as target sectors, the amount attributed to 236220 would not change, but rather 236115 and 236116 would each be attributed 4 (Box B in Fig. 5). In this way, the number of sectors that share a parent NAICS for a given sector length does not impact attribution to other “cousin” sectors. Eq. (2) provides the formula for equal allocation, where $n_{t}$ is the total number of relevant child sectors.

(2) $$e_{s}=e_{t}\frac{1}{n_{t}}$$

The national detail model (*m2*) attributes GHGs to more detailed sectors (540) than used in the 2020 NGIAM model, which attributed emissions to BEA Detail level industries (∼400). Because a primary use case for this dataset is for use in USEEIO models, previously the BEA Detail schema, which is the schema used by USEEIO v1 models, was used as the target industry classification set. However, BEA uses categories like *1111A0*, which include selected 5-digit NAICS codes within the 4-digit industry *1111*. We attribute to the more resolved NAICS codes, like *11111* and *11112*, that are associated with the aggregate BEA code. This results in a higher sector resolution.

Table 2 shows the data years used for attribution sources when necessary for the new models alongside the NGIAM year 2016 model for reference. Data from the latest available year of BEA Make and Use tables from BEA are used wherever the Make and Use tables are used as an allocation source. Because the official Detail input-output tables are only issued every 5 years, we imputed annual Detail tables using the monetary flows from the Summary tables and proportionally-allocated those across the respective detail sectors using most recent detail tables (2012 or 2017). This imputation is performed using the flow-by-sector methods, *Detail_Use.yaml* and *Detail_Supply.yaml*. A common year of data from the most recently published results of the quadrennial Manufacturing Energy Consumption Survey (MECS) 2018 [14], was used for 2016 and later year models, and the 2014 MECS data were used for all earlier models [15]. Similarly, the 2017 Census of Agriculture data [8,9] are used for years 2016 and beyond, while the 2012 data are used for prior years. The annual USGS Mineral Yearbook report for Lead [16] is used corresponding with the GHGI year, except for 2020, where 2019 data are used because they were the most recent available at the time of publication. The data acquisition dates can also result in changes if the allocation data provider revised the data source, even if revision information is not provided. The data sources shown in Table 2 in the NGIAM 2016 were acquired at different times than those used in the national aggregated model (*m1*).

**Table 2:** Data Years for Allocation Sources By Model and Year.

Allocation Source	2016 NGIAM	2012 m1	2013 m1	2014 m1	2015 m1	2016 m1	2017 m1	2018 m1	2019 m1	2020 m1
Detail Make	2012	2012	2013	2014	2015	2016	2017	2018	2019	2020
Detail Use	2012	2012	2013	2014	2015	2016	2017	2018	2019	2020
MECS Tables 2-2, 3-2	2014	2014	2014	2014	2014	2018	2018	2018	2018	2018
CoA Cropland	2017	2012	2012	2012	2012	2017	2017	2017	2017	2017
CoA Cropland NAICS	2017	2012	2012	2012	2012	2017	2017	2017	2017	2017
MYB Lead	2016	2012	2013	2014	2015	2016	2017	2018	2019	2019

### 4.2 Estimating suppressed data from EIA MECS

The EIA MECS reports energy use by 3- to 6-digit NAICS and fuel type, such as coal or natural gas. MECS data for select NAICS and fuels are suppressed because the estimated energy use is less than 0.5 trillion Btu, the value represents a single establishment, or because the energy estimate's relative standard error (RSE) is greater than 50% [14]. When the energy use is suppressed because the value is less than 0.5 trillion Btu, we estimate this suppressed data by assigning the NAICS a value of 0.25 trillion Btu. These estimates of suppressed data represent a small fraction of energy use by sectors. For example, coal consumption by industries 31-33 in 2018 was approximately 432 trillion Btu [14]. EIA MECS suppressed 19 sectors at various sector lengths for being less than 0.5 trillion Btu, which, at most, would represent 1.7% of national coal consumption. Values suppressed for any other reason are estimated by proportionally attributing any unallocated reported parent values to the suppressed child values using a secondary source, in this case, Detail Use data. For example, national coal consumption for NAICS 325211 and 325312 are suppressed because the values represent single establishments, but MECS publishes coal consumption by the parent NAICS 325. Any unallocated flows from the parent 325 are proportionally attributed to the suppressed child values, as demonstrated in Eq. (1).

### 4.3 Attributing industrial emissions to manufacturing sectors

Industrial stationary combustion emissions are sourced from GHGI Tables A-6, 3-8, 3-9. These tables report emissions by fuel and sector. In the GHGI, the “Industrial” sector includes manufacturing sectors (NAICS 31-33) but also non-manufacturing industrial sector such as Agriculture, Mining, and Construction. EIA MECS, the primary attribution source for industrial sources, only captures manufacturing sectors, so a second source is necessary for the non-manufacturing sectors. As a result, industrial emissions are first attributed between manufacturing and non-manufacturing sectors by comparing fuel use in EIA MECS with that reported in GHGI Table A-6 for all industrial sectors. For 2018, we estimate that 76% of coal consumption and 68% of natural gas consumption is from manufacturing sectors. The remaining emissions are attributed to non-manufacturing sectors and attributed within those sectors based on the Use table.

### 4.4 Estimating hydrofluorocarbons by species

Emissions of hydrofluorocarbons (HFCs) and perflurocarbons (PFCs) as ozone depleting substance (ODS) substitutes are sourced primarily from GHGI Table A-97 for transportation and GHGI Table 4-102 for other activities. These tables report HFCs and PFCs in aggregate (in units of CO2e). In alignment with the goal of reporting all flows in units of raw mass, the specific flow breakdown from these sources is estimated based on the emissions reported in GHGI Table 4-100, which includes the emissions of seven specific HFCs and PFCs from ODS Substitutes. The remaining non-specified flows (described as “Others” in this table) are maintained as “HFCs and PFCs, unspecified” in units of CO2e.

### 4.5 Description of Changes from 2020 NGIAM

Methodological updates were made to correct for errors in the previous version, to make use of higher resolution GHGI data now available, and to account for other corrections, data updates, and methodological updates. Notable revisions to emissions estimates in the GHGI since the publication of the NGHGIAM are also presented. A list of changes including subsections describing method changes, data updates, and corrections is included below.

### 4.6 Methodology changes

1. Map to NAICS industries in place of direct mapping of emissions to BEA Detail industries (described above).
2. Revise the flow used from EIA MECS to attribute stationary combustion emissions from petroleum products.
3. Revise the approach to impute missing or non-reported data by sector from EIA MECS.
4. Refine the attribution approach for HFCs and PFCs from ODS Substitutes (GHGI Table 4-102) to account for increased resolution of data reporting.
5. Simplify handling of gasoline and diesel fuel for transport allocation to be consistent with other Use table allocation methods.

### 4.7 Data updates

6. MECS: 2018 in place of 2014 data.
7. GHGI: Revisions in non-road and alternative fuel mobile combustion emissions for CH4 and N2O (GHGI Tables 3-14, 3-15).
8. GHGI: Revisions to agricultural N2O emissions, direct and indirect (GHGI Tables 5-17, 5-18).
9. GHGI: Increases in CH4 to emissions from wastewater and rice cultivation (GHGI Table 2-1).
10. GHGI: Revisions to emissions from natural gas systems (GHGI Table 3-68).
11. GHGI: Revisions to emissions from manure management (GHGI Table 5-6).
12. BEA Make and Use: Updated release of the BEA Detail Make and Use tables.
13. GHGI: Increase in N2O from stationary combustion for electric power for coal (GHGI Table 3-9).

### 4.8 Corrections

14. Fix error under attributing stationary combustion emissions to some sectors, notably 331110 and 325180.
15. Fix error mis-attributing petrochemical emissions to the wrong sectors (GHGI Table 4-46).

### 4.9 State GHG Models

The state level models rely on the national aggregate model as the primary emissions source. For attribution to specific states, they use EPA's Inventory of U.S. Greenhouse Gas Emissions and Sinks by State (State GHGI), a recently developed dataset that is meant to provide state level estimates of GHG emissions that aligns with the national GHGI [17]. State level datasets are presented here for the first time, as they were not present in the NGIAM. The national aggregate model is chosen as a starting point for the state models, rather than the State GHGI. The national model provides additional detail in some cases that make it more appropriate as a starting place and assures that state totals aggregate to national totals for each sector. Like the national inventory, the state inventories provide data annually from 1990-2020 and the full range of GHGs. Because the structures of the source datasets (or FBAs) are not the same between the state and national inventories, additional Activity-to-Sector mapping files are required for the Greenhouse Gas Totals By Summary Industry by State dataset described here.

As shown in Fig. 4, the state model uses the aggregate national FBS as its starting point. National emissions by sector are attributed across all states proportionally based on the emissions reported for specific activities in the State GHGI. In select cases, where the granularity of the State GHGI is less than that in the national GHGI, emissions are instead attributed using state-level Make and Use tables from the State IO models [18]. For example, mobile source CO2 emissions from aircraft and boats are attributed across states based on the use of petroleum fuels by sectors 481 and 483, respectively, instead of based on reported CO2 emissions in the State GHGI, for which the most granular emission activity is “Transportation - CO2 from Fossil Fuel Combustion - Fossil Fuel Combustion - Petroleum.” Using this single activity from the State GHGI as an attribution source would yield emissions for aircraft and boats in the same ratio across all states, a result unlikely to be accurate. In this way, the state model can reflect state-specific consumption of commodities by industries as a means of attribution.

### 4.10 Technical Validation

We validate the datasets though comparisons to reference GHG totals from the EPA GHGI and state GHGI, compare to the NGIAM output, and we compare the total across the state datasets to our national totals.

### 4.11 Comparison to Reported Totals

Table 3 presents the results of the validation check against the GHGI totals by gas or groups of minor gas. All differences can be explained by rounding errors, except for the HFCs, due to the lack of attribution of some activities to industries with HFC emissions. Rounding errors occur due to two reasons within the GHGI: 1) totals of line items may not add to respective subtotals or grand totals due to lack of precision, and 2) in a number of tables a ‘+’ symbol is present indicating that GHG emissions are present but at a smaller quantity than can be represented as non-zero by the decimal unit in the table.

**Table 3:** Relative difference between sum of GHG or groups between the national aggregate model (m1) and GHGI.

GHG	2012	2013	2014	2015	2016	2017	2018	2019	2020
Carbon dioxide	1.003	1.002	1.002	1.002	1.001	1.001	1.000	0.999	0.999
HFCs	1.001	1.002	1.001	1.001	1.001	1.002	1.003	1.003	1.002
Methane	0.997	0.997	0.998	0.997	0.997	0.997	0.997	0.997	0.998
Nitrogen trifluoride	1.000	1.000	1.000	1.000	1.000	1.000	1.000	1.000	1.000
Nitrous oxide	0.995	0.997	0.995	0.996	0.996	0.997	0.996	0.996	0.996
PFCs	1.000	0.985	0.990	0.981	1.002	0.974	1.008	1.013	1.041
Sulfur hexafluoride	1.000	1.000	1.000	1.000	1.000	1.000	0.982	1.000	1.000
total CO2e	1.002	1.002	1.001	1.001	1.001	1.000	1.000	0.999	0.999

### 4.12 Comparison to Previous Model

High-resolution images are provided in the supporting figures that show the relative changes in the results by sector for each major GHG and for minor gases in CO2e [11]. For this analysis, the NGIAM 2016 model emissions are set to 1 for a given sector, and all other models results are shown as relative changes in comparison with this model. Table 4 provides a summary of significant data changes by sector for each gas and the associated explanation from the list above.

**Table 4:** Noteworthy changes in 2016 emissions with model update. D = Data update, M = Methodology update, C = Correction. The “Reason #” refers to one or more changes in the Description of Changes section above.

GHG(s)	Industries (Codes)	Reason Type	Reason #
All Major GHGs	111400, 111900, 112A00	D	12
All Major GHGs	23*	M	1
All Major GHGs	31*-33*, notably 325110, 325120, 325211	M	2
All Major GHGs	31*-33*	D, M	3, 6
All Major GHGs	325180, 331110	C	14
All Major GHGs	531*	M	1
CO2	212100	D	6
CO2	3252A0, 325411, 325180, 325190	C	15
CO2	491000, 492000	M	5
CH4	1111B0	D	9
CH4	112*	D	11
CH4	211000, 221120, 486000	D	10
CH4	221130	D	9
CH4	21*, 485000, 48A000, 492000, 532100, 532400, 621900, 713900, 811100, 811300	D	7
N2O	111*, 112*	D	8
N2O	11*, 21*,	D	7
N2O	2211	D	13
N2O	221300	D	9
N2O	485, 48A, 492, 5321, 5324, 6219, 7139, 8111, 8113	D	6
Other GHGs	All	M	4

### 4.13 Comparison Across Years

The change in emissions by sector across years is generally very minor from 2016 to 2020 and less significant than the difference between 2016 in the old and new models. Images are provided in the supporting figures [11]. There is frequently a steady increase in totals by sectors from 2016 to 2019 which then falls back in 2020. This mirrors the change in the GHGI over this period for industry for what are defined in the GHGI as industry, transportation, agriculture, residential and commercial sectors (GHGI Table 2-1). GHG emissions from electricity are an exception to this trend, and they decrease steadily through the entire period, with a larger drop from 2019 to 2020.

### 4.14 State comparison to National

As a form of validation and review of the state GHG model, we confirmed that, for all GHGs:

1. The sum totals for a given GHG and sector across all states (and the District of Columbia) equals the national total for the GHG and sector, as in Eq. (3), where e represents a vector of GHG totals, s is a sector, f is a flow, st is a state, and n is the nation.
  (3) $$\sum_{st=1}^{51}e_{s,f,st}=e_{s,f,n}$$
2. The sum totals for a given GHG across all states equals the national total for the given GHG, as in Eq. (4).
  (4) $$\sum_{st=1}^{51}e_{f,st}=e_{f,n}$$

The national totals for these validation tests were derived from the aggregate level model, where the sectors match the state model. We performed this test for each year of data from 2012-2020. The result of the test in Eq. (3) show that state totals are <1% different than national totals for all gases and sectors in all years with some exceptions for the smaller quantity GHGs (HFCs, CF4, SF6, NF3, etc.) in the equipment sectors, like primary metal manufacturing. The result of the second test in Eq. (4) showed that these differences are cancelled out when combining sectors, such that totals by gas across all states equal those at the national level. The only exception to the second test is when the reported precision for emissions of small quantities in the national inventory (e.g., for Perfluorocyclobutane and Perfluoropropane) is insufficient when compared to the data in the state GHG inventories. Future work will seek to resolve these data precision issues with the data providers.

### 4.15 State comparison to EPA's Inventory

**Fig. 6:**
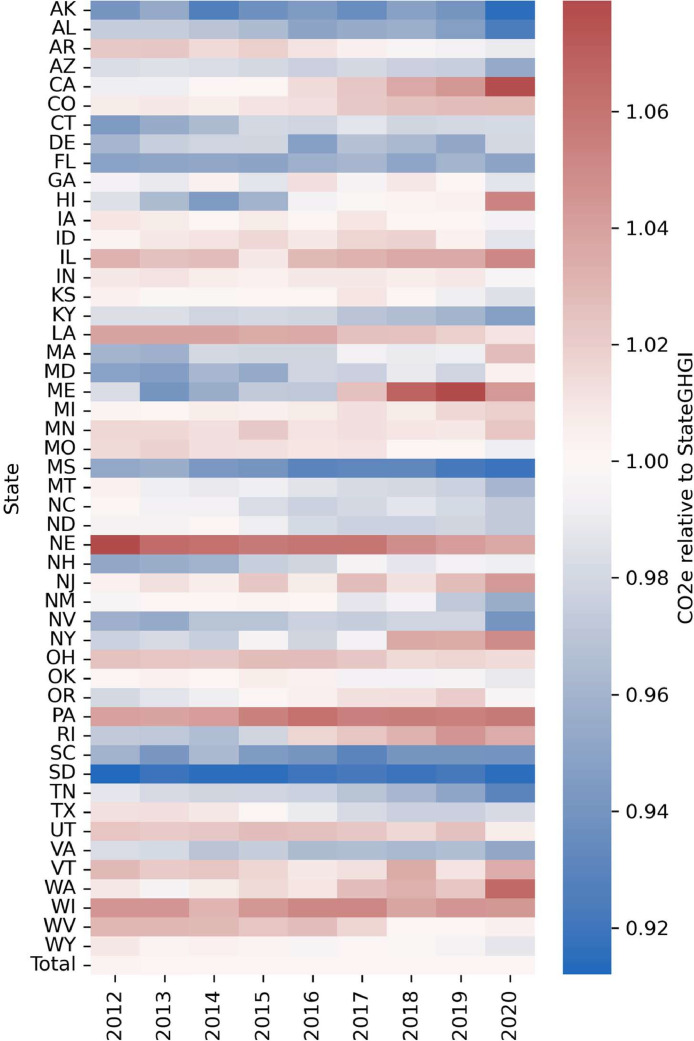
Comparison of modeled emissions (CO2e) to reported state totals.

In developing the state model, we use the State GHGI as an attribution source and not a primary emission source in order to ensure that the sum of all state totals by sector is equal to the national totals by sector (see previous section). Therefore, here we compare the modeled state totals to the reported emissions in EPA's State GHGI in Fig. 6. All state totals are within +/- 10% of emissions reported in the State GHGI, with most states yielding much closer results.

### 4.16 Code Availability

The models used to produce the datasets are incorporated into FLOWSA v2.0.0 and available in the FLOWSA source code repository. The model code is represented in a number of YAML files that can be found in the *flowsa/methods/flowbysector* folder. *GHG_national_m1_common* is national aggregate common model with specific implementations for each year from 2012-2020. *GHG_state_m1* is the state aggregate common model with specific implementations for each year from 2012-2020. *GHG_national_m2* is the national detail common model with specific implementations for each year from 2012-2020. The specific GHGI-to-NAICS crosswalk file can be found in *flowsa/data/activitytosectormapping*. Instructions in the repository README and the associated FLOWSA Wiki describe how flow-by-sector methods can be executed.

## Limitations

The data represent emissions estimates from models and not real measurements. Limitations of estimates from the U.S. GHG Inventory and State Inventories apply to these datasets. The choice of datasets for attribution are subject to interpretation and are limited by data availability. Emissions from U.S. Territories that are included in the GHGI are excluded here, due to a difference in geographical boundary between the GHGI and this dataset. Biogenic carbon emissions, or emissions from the combustion or decay of wood or other bio-based materials, are excluded just as they are excluded UNFCCC national inventory emissions. HFCs and PFCs related to solvent use, aerosols, and fire protection are not included, due to lack of data for sector attribution. These emissions represent ∼1% of total HFC and PFC emissions reported in the GHGI. Some HFCs and PFCs are reported as a total CO2e and not by individual species; due to a lack of species resolution.

## Ethics Statement

The authors have read and follow the ethical requirements for publication in Data in Brief and confirm that the current work does not involve human subjects, animal experiments, or any data collected from social media platforms.

## CRediT authorship contribution statement

**Ben Young:** Methodology, Software, Validation, Data curation, Writing – original draft, Visualization. **Catherine Birney:** Methodology, Software, Visualization. **Wesley W. Ingwersen:** Conceptualization, Methodology, Writing – original draft, Supervision.

## Figures and Tables

**Table 5:** Attributions by Activity for national aggregate model (m1).

Source	Activity	Allocation Source	Sectors
EPA_GHGI_T_2_1	Abandoned Oil and Gas Wells		211
EPA_GHGI_T_2_1	Abandoned Underground Coal Mines		2121
EPA_GHGI_T_2_1	Adipic Acid Production		32519
EPA_GHGI_T_2_1	Aluminum Production		331313
EPA_GHGI_T_2_1	Ammonia Production		32531
EPA_GHGI_T_2_1	Anaerobic Digestion at Biogas Facilities		562219
EPA_GHGI_T_2_1	Caprolactam, Glyoxal, and Glyoxylic Acid Production		32519
EPA_GHGI_T_2_1	Carbide Production and Consumption		32518
EPA_GHGI_T_2_1	Cement Production		32731
EPA_GHGI_T_2_1	Coal Mining		2121
EPA_GHGI_T_2_1	Composting		562219
EPA_GHGI_T_2_1	Ferroalloy Production		3311
EPA_GHGI_T_2_1	Glass Production		3272
EPA_GHGI_T_2_1	Landfills		562212
EPA_GHGI_T_2_1	Lime Production		3274
EPA_GHGI_T_2_1	Incineration of Waste		562213
EPA_GHGI_T_2_1	Iron and Steel Production & Metallurgical Coke Production		3311
EPA_GHGI_T_2_1	Nitric Acid Production		32531
EPA_GHGI_T_2_1	Phosphoric Acid Production		32531
EPA_GHGI_T_2_1	Rice Cultivation		11116
EPA_GHGI_T_2_1	Soda Ash Production		32519
EPA_GHGI_T_2_1	Titanium Dioxide Production		32519
EPA_GHGI_T_2_1	Wastewater Treatment		2213
EPA_GHGI_T_2_1	Zinc Production		33142
EPA_GHGI_T_2_1	Electrical Transmission and Distribution		2211
EPA_GHGI_T_2_1	Electric Power	BEA_Make_Detail_BeforeRedef	2211, S00101, S00202
EPA_GHGI_T_2_1	Liming	BEA_Use_Detail_PRO_BeforeRedef	111, 112
EPA_GHGI_T_2_1	Urea Fertilization	BEA_Use_Detail_PRO_BeforeRedef	111, 112
EPA_GHGI_T_2_1	Urea Consumption for Non-Agricultural Purposes	BEA_Use_Detail_PRO_BeforeRedef	31, 32, 33
EPA_GHGI_T_2_1	Other Process Uses of Carbonates	BEA_Use_Detail_PRO_BeforeRedef	31, 32, 33
EPA_GHGI_T_2_1	Lead Production	USGS_MYB_Lead	331410, 33149
EPA_GHGI_T_2_1	N2O from Product Uses	BEA_Use_Detail_PRO_BeforeRedef	621, 622, 623
EPA_GHGI_T_3_68	Distribution		221210
EPA_GHGI_T_3_68	Distribution - Post-Meter		221210
EPA_GHGI_T_3_68	Exploration		211111
EPA_GHGI_T_3_68	Processing		211111
EPA_GHGI_T_3_68	Production		211111
EPA_GHGI_T_3_68	Transmission and Storage		486210
EPA_GHGI_T_3_70	Distribution		221210
EPA_GHGI_T_3_70	Distribution - Post-Meter		221210
EPA_GHGI_T_3_70	Exploration		211111
EPA_GHGI_T_3_70	Processing		211111
EPA_GHGI_T_3_70	Production		211111
EPA_GHGI_T_3_70	Transmission and Storage		486210
EPA_GHGI_T_3_72	Distribution		221210
EPA_GHGI_T_3_72	Distribution - Post-Meter		221210
EPA_GHGI_T_3_72	Exploration		211111
EPA_GHGI_T_3_72	Processing		211111
EPA_GHGI_T_3_72	Production		211111
EPA_GHGI_T_3_72	Transmission and Storage		486210
EPA_GHGI_T_3_42	Crude Oil Transportation		486110
EPA_GHGI_T_3_42	Transportation		486110
EPA_GHGI_T_3_42	Exploration		211111
EPA_GHGI_T_3_42	Production		211111
EPA_GHGI_T_3_42	Refining		324110
EPA_GHGI_T_3_42	Crude Refining		324110
EPA_GHGI_T_3_44	Crude Oil Transportation		486110
EPA_GHGI_T_3_44	Transportation		486110
EPA_GHGI_T_3_44	Exploration		211111
EPA_GHGI_T_3_44	Production		211111
EPA_GHGI_T_3_44	Refining		324110
EPA_GHGI_T_3_44	Crude Refining		324110
EPA_GHGI_T_3_46	Crude Oil Transportation		486110
EPA_GHGI_T_3_46	Transportation		486110
EPA_GHGI_T_3_46	Exploration		211111
EPA_GHGI_T_3_46	Production		211111
EPA_GHGI_T_3_46	Refining		324110
EPA_GHGI_T_3_46	Crude Refining		324110
EPA_GHGI_T_5_28	Chickpeas		11113
EPA_GHGI_T_5_28	Cotton		11192
EPA_GHGI_T_5_28	Maize		11115
EPA_GHGI_T_5_28	Rice		11116
EPA_GHGI_T_5_28	Soybeans		11111
EPA_GHGI_T_5_28	Wheat		11114
EPA_GHGI_T_5_3	American Bison		11299
EPA_GHGI_T_5_3	Beef Cattle		11211
EPA_GHGI_T_5_3	Dairy Cattle		11212
EPA_GHGI_T_5_3	Goats		11242
EPA_GHGI_T_5_3	Horses		11292
EPA_GHGI_T_5_3	Mules and Asses		11292
EPA_GHGI_T_5_3	Sheep		11241
EPA_GHGI_T_5_3	Swine		1122
EPA_GHGI_T_5_3	Poultry		1123
EPA_GHGI_T_5_6	American Bison		11299
EPA_GHGI_T_5_6	Beef Cattle		11211
EPA_GHGI_T_5_6	Dairy Cattle		11212
EPA_GHGI_T_5_6	Goats		11242
EPA_GHGI_T_5_6	Horses		11292
EPA_GHGI_T_5_6	Mules and Asses		11292
EPA_GHGI_T_5_6	Sheep		11241
EPA_GHGI_T_5_6	Swine		1122
EPA_GHGI_T_5_6	Poultry		1123
EPA_GHGI_T_5_17	Organic Amendment Cropland	BEA_Use_Detail_PRO_BeforeRedef	111
EPA_GHGI_T_5_17	Residue N Cropland	BEA_Use_Detail_PRO_BeforeRedef	111
EPA_GHGI_T_5_17	Synthetic Fertilizer Cropland	BEA_Use_Detail_PRO_BeforeRedef	111
EPA_GHGI_T_5_17	Mineralization and Asymbiotic Fixation Cropland	USDA_CoA_Cropland	1111, 1112, 1113, 1119
EPA_GHGI_T_5_17	Drained Organic Soils Cropland	USDA_CoA_Cropland	1111, 1112, 1113, 1119
EPA_GHGI_T_5_17	All activities Grassland	USDA_CoA_Cropland_NAICS	112
EPA_GHGI_T_5_18	Volatilization & Atm. Deposition Cropland	BEA_Use_Detail_PRO_BeforeRedef	111
EPA_GHGI_T_5_18	Surface Leaching & Run-Off Cropland	BEA_Use_Detail_PRO_BeforeRedef	111
EPA_GHGI_T_5_18	All activities Grassland	USDA_CoA_Cropland_NAICS	112
EPA_GHGI_T_3_13	General Aviation Aircraft		481
EPA_GHGI_T_3_13	Commercial Aircraft		481
EPA_GHGI_T_3_13	Ships and Non-Recreational Boats		483
EPA_GHGI_T_3_13	Light-Duty Trucks - Households		F01000
EPA_GHGI_T_3_13	Motorcycles		F01000
EPA_GHGI_T_3_13	Military Aircraft		928110
EPA_GHGI_T_3_13	Rail		482
EPA_GHGI_T_3_13	Medium- and Heavy-Duty Trucks		484
EPA_GHGI_T_3_13	Passenger Cars - Households		F01000
EPA_GHGI_T_3_13	Recreational Boats		F01000
EPA_GHGI_T_3_13	Pipeline Natural Gas		486
EPA_GHGI_T_3_13	Buses		485
EPA_GHGI_T_3_13	Passenger Cars	BEA_Use_Detail_PRO_BeforeRedef	491, 922, F01000
EPA_GHGI_T_3_13	Light-Duty Trucks	BEA_Use_Detail_PRO_BeforeRedef	492, F01000
EPA_GHGI_T_3_13	Buses - Distillate Fuel Oil	BEA_Use_Detail_PRO_BeforeRedef	485, 487, 488, S00201
EPA_GHGI_T_3_13	Medium- and Heavy-Duty Trucks - Distillate Fuel Oil	BEA_Use_Detail_PRO_BeforeRedef	484, 92212, 92214, 92216
EPA_GHGI_T_3_14	General Aviation Aircraft		481
EPA_GHGI_T_3_14	Ships and Boats		483
EPA_GHGI_T_3_14	Rail		482
EPA_GHGI_T_3_14	Medium- and Heavy-Duty Trucks and Buses		484
EPA_GHGI_T_3_14	Motorcycles		F01000
EPA_GHGI_T_3_14	Passenger Cars	BEA_Use_Detail_PRO_BeforeRedef	491, 922, F01000
EPA_GHGI_T_3_14	Light-Duty Trucks	BEA_Use_Detail_PRO_BeforeRedef	492, F01000
EPA_GHGI_T_3_14	Buses - Distillate Fuel Oil	BEA_Use_Detail_PRO_BeforeRedef	485, 487, 488, S00201
EPA_GHGI_T_3_14	Medium- and Heavy-Duty Trucks - Distillate Fuel Oil	BEA_Use_Detail_PRO_BeforeRedef	484, 92212, 92214, 92216
EPA_GHGI_T_3_14	Construction/Mining Equipment Non-Road	BEA_Use_Detail_PRO_BeforeRedef	211, 212, 213111
EPA_GHGI_T_3_14	Agricultural Equipment Non-Road	BEA_Use_Detail_PRO_BeforeRedef	111, 112, 113, 114
EPA_GHGI_T_3_14	Other Non-Road	BEA_Use_Detail_PRO_BeforeRedef	485, 487, 488, 492, 5321, 5324, 6219, 7139, 8111, 8113
EPA_GHGI_T_3_14	Alternative Fuel On-Road	BEA_Use_Detail_PRO_BeforeRedef	485, 487, 488, 492
EPA_GHGI_T_3_15	General Aviation Aircraft		481
EPA_GHGI_T_3_15	Ships and Boats		483
EPA_GHGI_T_3_15	Rail		482
EPA_GHGI_T_3_15	Medium- and Heavy-Duty Trucks and Buses		484
EPA_GHGI_T_3_15	Motorcycles		F01000
EPA_GHGI_T_3_15	Passenger Cars	BEA_Use_Detail_PRO_BeforeRedef	491, 922, F01000
EPA_GHGI_T_3_15	Light-Duty Trucks	BEA_Use_Detail_PRO_BeforeRedef	492, F01000
EPA_GHGI_T_3_15	Buses - Distillate Fuel Oil	BEA_Use_Detail_PRO_BeforeRedef	485, 487, 488, S00201
EPA_GHGI_T_3_15	Medium- and Heavy-Duty Trucks - Distillate Fuel Oil	BEA_Use_Detail_PRO_BeforeRedef	484, 92212, 92214, 92216
EPA_GHGI_T_3_15	Construction/Mining Equipment Non-Road	BEA_Use_Detail_PRO_BeforeRedef	211, 212, 213111
EPA_GHGI_T_3_15	Agricultural Equipment Non-Road	BEA_Use_Detail_PRO_BeforeRedef	111, 112, 113, 114
EPA_GHGI_T_3_15	Other Non-Road	BEA_Use_Detail_PRO_BeforeRedef	485, 487, 488, 492, 5321, 5324, 6219, 7139, 8111, 8113
EPA_GHGI_T_3_15	Alternative Fuel On-Road	BEA_Use_Detail_PRO_BeforeRedef	485, 487, 488, 492
EPA_GHGI_T_4_46	Acrylonitrile		325199
EPA_GHGI_T_4_46	Carbon Black		325180
EPA_GHGI_T_4_46	Ethylene		324110
EPA_GHGI_T_4_46	Ethylene Dichloride		325120
EPA_GHGI_T_4_46	Ethylene Oxide		325199
EPA_GHGI_T_4_46	Methanol		325199
EPA_GHGI_T_4_50	HCFC-22 Production		325120
EPA_GHGI_T_4_96	Electronics Production		334413
EPA_GHGI_T_4_102	Domestic Refrigeration		F01000
EPA_GHGI_T_4_102	Residential Stationary Air Conditioning		F01000
EPA_GHGI_T_4_102	Commercial Refrigeration	BEA_Use_Detail_PRO_BeforeRedef	2213, 42, 44, 45, 493, 51, 52, 53, 54, 55, 56, 61, 62, 71, 72, 81, 923110, 923120, 927110
EPA_GHGI_T_4_102	Industrial Process Refrigeration	BEA_Use_Detail_PRO_BeforeRedef	11, 21, 2212, 23, 31, 32, 33
EPA_GHGI_T_4_102	Commercial Stationary Air Conditioning	BEA_Use_Detail_PRO_BeforeRedef	2213, 42, 44, 45, 493, 51, 52, 53, 54, 55, 56, 61, 62, 71, 72, 81, 923110, 923120, 927110
EPA_GHGI_T_4_102	Foams	BEA_Use_Detail_PRO_BeforeRedef	11, 21, 22, 23, 31, 32, 33, 42, 44, 45, 48, 49, 51, 52, 53, 54, 55, 56, 61, 62, 71, 72, 81, 92, F01000
EPA_GHGI_T_A_97	Light-Duty Trucks - Households		F01000
EPA_GHGI_T_A_97	Heavy-Duty Vehicles		484
EPA_GHGI_T_A_97	Ships and Boats		483
EPA_GHGI_T_A_97	School and Tour Buses		485
EPA_GHGI_T_A_97	Rail		482
EPA_GHGI_T_A_97	Medium- and Heavy-Duty Trucks		484
EPA_GHGI_T_A_97	Passenger Cars - Households		F01000
EPA_GHGI_T_4_80	Aluminum Production		331313
EPA_GHGI_T_4_86	Magnesium Production and Processing	BEA_Make_Detail_BeforeRedef	331410, 33149, 33152
EPA_GHGI_T_3_22	Industry Industrial Coking Coal		212210
EPA_GHGI_T_3_22	Industry Industrial Other Coal		212210
EPA_GHGI_T_3_22	Industry Petroleum Products Non-energy	EIA_MECS_Energy	31, 324110, 324199, 325, 326, 327, 33
EPA_GHGI_T_3_22	Industry Natural Gas to Chemical Plants	EIA_MECS_Energy	31, 32, 33
EPA_GHGI_T_3_22	Transportation Lubricants	BEA_Use_Detail_PRO_BeforeRedef	48, 491, 492, 92, F01000
EPA_GHGI_T_A_5	Total (All Fuels) Residential		F01000
EPA_GHGI_T_A_5	Coal Industrial	BEA_Use_Detail_PRO_BeforeRedef	11, 21, 2212, 23
EPA_GHGI_T_A_5	Coal Commercial	BEA_Use_Detail_PRO_BeforeRedef	2213, 42, 44, 45, 493, 51, 52, 53, 54, 55, 56, 61, 62, 71, 72, 81, 923110, 923120, 927110
EPA_GHGI_T_A_5	Natural Gas Commercial	BEA_Use_Detail_PRO_BeforeRedef	2213, 42, 44, 45, 493, 51, 52, 53, 54, 55, 56, 61, 62, 71, 72, 81, 923110, 923120, 927110
EPA_GHGI_T_A_5	Natural Gas Industrial	BEA_Use_Detail_PRO_BeforeRedef	11, 21, 2212, 23
EPA_GHGI_T_A_5	Coal Industrial - Manufacturing	EIA_MECS_Energy	31, 32, 33
EPA_GHGI_T_A_5	Natural Gas Industrial - Manufacturing	EIA_MECS_Energy	31, 32, 33
EPA_GHGI_T_A_5	Total Petroleum Commercial	BEA_Use_Detail_PRO_BeforeRedef	2213, 42, 44, 45, 493, 51, 52, 53, 54, 55, 56, 61, 62, 71, 72, 81, 923110, 923120, 927110
EPA_GHGI_T_A_5	Total Petroleum Industrial	BEA_Use_Detail_PRO_BeforeRedef	11, 21, 2212, 23, 31, 32, 33
EPA_GHGI_T_3_8	Fuel Oil Residential		F01000
EPA_GHGI_T_3_8	Coal Residential		F01000
EPA_GHGI_T_3_8	Natural Gas Residential		F01000
EPA_GHGI_T_3_8	Wood Residential		F01000
EPA_GHGI_T_3_8	Natural Gas Electric Power	BEA_Make_Detail_BeforeRedef	2211, S00101, S00202
EPA_GHGI_T_3_8	Fuel Oil Electric Power	BEA_Make_Detail_BeforeRedef	2211, S00101, S00202
EPA_GHGI_T_3_8	Wood Electric Power	BEA_Make_Detail_BeforeRedef	2211, S00101, S00202
EPA_GHGI_T_3_8	Coal Electric Power	BEA_Make_Detail_BeforeRedef	2211, S00101, S00202
EPA_GHGI_T_3_8	Fuel Oil Commercial	BEA_Use_Detail_PRO_BeforeRedef	2213, 42, 44, 45, 493, 51, 52, 53, 54, 55, 56, 61, 62, 71, 72, 81, 923110, 923120, 927110
EPA_GHGI_T_3_8	Fuel Oil Industrial	BEA_Use_Detail_PRO_BeforeRedef	11, 21, 2212, 23, 31, 32, 33
EPA_GHGI_T_3_8	Natural Gas Industrial	BEA_Use_Detail_PRO_BeforeRedef	11, 21, 2212, 23
EPA_GHGI_T_3_8	Natural Gas Commercial	BEA_Use_Detail_PRO_BeforeRedef	2213, 42, 44, 45, 493, 51, 52, 53, 54, 55, 56, 61, 62, 71, 72, 81, 923110, 923120, 927110
EPA_GHGI_T_3_8	Coal Industrial	BEA_Use_Detail_PRO_BeforeRedef	11, 21, 2212, 23
EPA_GHGI_T_3_8	Coal Industrial - Manufacturing	EIA_MECS_Energy	31, 32, 33
EPA_GHGI_T_3_8	Natural Gas Industrial - Manufacturing	EIA_MECS_Energy	31, 32, 33
EPA_GHGI_T_3_9	Fuel Oil Residential		F01000
EPA_GHGI_T_3_9	Coal Residential		F01000
EPA_GHGI_T_3_9	Natural Gas Residential		F01000
EPA_GHGI_T_3_9	Wood Residential		F01000
EPA_GHGI_T_3_9	Natural Gas Electric Power	BEA_Make_Detail_BeforeRedef	2211, S00101, S00202
EPA_GHGI_T_3_9	Fuel Oil Electric Power	BEA_Make_Detail_BeforeRedef	2211, S00101, S00202
EPA_GHGI_T_3_9	Wood Electric Power	BEA_Make_Detail_BeforeRedef	2211, S00101, S00202
EPA_GHGI_T_3_9	Coal Electric Power	BEA_Make_Detail_BeforeRedef	2211, S00101, S00202
EPA_GHGI_T_3_9	Fuel Oil Commercial	BEA_Use_Detail_PRO_BeforeRedef	2213, 42, 44, 45, 493, 51, 52, 53, 54, 55, 56, 61, 62, 71, 72, 81, 923110, 923120, 927110
EPA_GHGI_T_3_9	Fuel Oil Industrial	BEA_Use_Detail_PRO_BeforeRedef	11, 21, 2212, 23, 31, 32, 33
EPA_GHGI_T_3_9	Natural Gas Industrial	BEA_Use_Detail_PRO_BeforeRedef	11, 21, 2212, 23
EPA_GHGI_T_3_9	Natural Gas Commercial	BEA_Use_Detail_PRO_BeforeRedef	2213, 42, 44, 45, 493, 51, 52, 53, 54, 55, 56, 61, 62, 71, 72, 81, 923110, 923120, 927110
EPA_GHGI_T_3_9	Coal Industrial	BEA_Use_Detail_PRO_BeforeRedef	11, 21, 2212, 23
EPA_GHGI_T_3_9	Coal Industrial - Manufacturing	EIA_MECS_Energy	31, 32, 33
EPA_GHGI_T_3_9	Natural Gas Industrial - Manufacturing	EIA_MECS_Energy	31, 32, 33

## Data Availability

2012-2020 Greenhouse Gas National- and State-Level Emission Totals by Industry (Original data) (Data.gov) U.S. and State GHG emissions by sector (2012-2020) - Supporting Figures (Original data) (figshare)

## Appendix

### Annotation of National Aggregate model YAML (*GHG_national_m1_common*)

The following excerpt is from the main instructions file, *GHG_national_m1_common.yaml*, that defines the general method used for national aggregate sector attribution of GHGs. This excerpt specifies use of selected data from the GHG Table 2-1.

!include:USEEIO_summary_target.yaml

target_naics_year: 2012

geoscale: national

···

source_names:

  EPA_GHGI_T_2_1:

    fedefl_mapping: GHGI

    activity_sets:

      direct:

        selection_fields:

          PrimaryActivity:

          - Abandoned Oil and Gas Wells

          - Abandoned Underground Coal Mines

          - Adipic Acid Production

          - Aluminum Production

          - Ammonia Production

          - Anaerobic Digestion at Biogas Facilities

          - Caprolactam, Glyoxal, and Glyoxylic Acid Production

          - Carbide Production and Consumption

          - Cement Production

          - Coal Mining

          - Composting

          - Ferroalloy Production

          - Glass Production

          - Landfills

          - Lime Production

          - Incineration of Waste

          - Iron and Steel Production & Metallurgical Coke Production

          - Nitric Acid Production

          - Phosphoric Acid Production

          - Rice Cultivation

          - Soda Ash Production

          - Titanium Dioxide Production

          - Wastewater Treatment

          - Zinc Production

           FlowName: [“CO2,” “CH4,” “N2O”] # HFCs and other flows are pulled elsewhere

     electricity_transmission:

        selection_fields:

          PrimaryActivity: Electrical Transmission and Distribution #SF6

          FlowName: SF6

        attribution_method: direct

      electric_power:

        selection_fields:

          PrimaryActivity:

            Electric Power Sector: Electric Power #CO2

        attribution_method: proportional

        attribution_source:

          Detail_Supply_YEAR: ## Update year

            selection_fields:

              SectorConsumedBy: {'2211′: ''}  # Electricity (221100)

      liming:

        selection_fields:

          PrimaryActivity: Liming #CO2

        attribution_method: proportional

        attribution_source:

          Detail_Use_YEAR: ## Update year

            selection_fields:

              SectorProducedBy: {'3274′: ''} # Lime (327400)

The first lines specify the target level of sectors in the result, classification system used and geographic scale for this method. These targets apply to all attribution that is dictated in later statements. The flow source *EPA_T_2_1* is specified under a *source_name*. A mapping file to map the flow names and units to the FEDEFL is given under *fedefl_mapping*, which corresponds to this mapping file name contained within the *fedelemflowlist* package. Multiple sets of activities (called activity sets) can be specified from a given source; in this case one set of activities are given under *direct* that are from the *EPA_T_2_1* source. Specific flows and activity names are selected for each activity set using the *selection_fields* tag. In this first example, this attribution applies to three source flows *CO2, CH4, N2O*. All emissions are directly attributed to NAICS sector per the allocation method. The third activity set is *electric_power. electric_power* uses electricity production from the Detail Supply table to further allocate it proportionally based on the production amounts in dollars from sectors that produce that commodity. Only sectors that consume electricity (“221100”) are included as a data point for calculating attribution. Notably, the year of the attribution source does not necessarily need to match the year of the primary data. The final activity set shown is *liming* of agricultural soils. A proportional allocation approach based on the dollars consumed of lime from the Detail Use table is used with a similar set of instructions as in the previous allocation.
